# A comprehensive in vitro comparison of the biological and physicochemical properties of bioactive root canal sealers

**DOI:** 10.1007/s00784-022-04570-2

**Published:** 2022-06-03

**Authors:** Sabina Noreen Wuersching, Christian Diegritz, Reinhard Hickel, Karin Christine Huth, Maximilian Kollmuss

**Affiliations:** Department of Conservative Dentistry and Periodontology, University Hospital, LMU Munich, Goethestrasse 70, 80336 Munich, Germany

**Keywords:** Antimicrobial properties, Apical periodontitis, Bioactivity, Cytotoxicity, Physicochemical properties, Root canal sealer

## Abstract

**Objectives:**

To evaluate the biological and physicochemical features of bioactive root canal sealers.

**Materials and methods:**

Human periodontal ligament fibroblasts (hPDLF) and human osteoblasts (hOB) were exposed to eluates of three bioactive root canal sealers, GuttaFlow® bioseal (GF), BioRoot™ RCS (BR), and TotalFill® BC Sealer (TF), and the epoxy resin–based sealer AH plus® (AH). Cytotoxicity and cellular inflammatory response were evaluated. The osteogenic potential was examined using human mesenchymal stem cells (hMSC). Film thickness, flowability, and pH were assessed. Root canal treatment was performed on human extracted teeth to evaluate the sealers’ tightness towards bacterial penetration. The antibacterial activity against common pathogens in primary root canal infections was tested.

**Results:**

AH was severely cytotoxic to hPDLF and hOB (*p* < 0.001). The bioactive sealers were generally less cytotoxic. IL-6 levels in hPDLF were elevated in the presence of AH (*p* < 0.05). AH and GF suppressed IL-6 production in hOB (*p* < 0.05). AH and BR stimulated the PGE_2_ production in hPDLF and hOB (*p* < 0.05). BR was the only sealer that led to calcium deposits in hMSC (*p* < 0.05). TF and AH showed the lowest film thickness and the highest flowability. Bacterial tightness was best in teeth filled with AH and BR. All sealers showed similar antimicrobial activity, but the overall antimicrobial efficacy was moderate as the bacteria were reduced by just one log scale (*p* < 0.05).

**Conclusions:**

This study revealed favorable in vitro results regarding the biocompatibility of the bioactive root canal sealers.

**Clinical relevance:**

Bioactive root canal sealers may be a useful alternative to epoxy resin–based sealers.

**Supplementary Information:**

The online version contains supplementary material available at 10.1007/s00784-022-04570-2.

## Introduction

Standard endodontic treatment of teeth with apical periodontitis (AP) requires a sufficient preparation of the root canal system to remove the infected pulp tissue, followed by a tight apical and coronal seal [1]. The current gold standard for root canal filling materials involves the use of a (semi-) solid material such as gutta-percha in combination with a root canal sealer (RCS) to fill the space between the gutta-percha and the root canal wall [2]. It has been reported that persistent AP occurs in 15–20% of the teeth treated due to primary AP, even when the endodontic procedure followed acceptable standards and the root canal filling appears sufficient in the radiograph [3–5]. One of the main causes for persistent AP are residual microorganisms grouped in biofilms which have not been entirely eliminated from the root canal [6]. Despite mechanical instrumentation and thorough disinfection of the pulp cavity, sufficient biofilm removal is not always possible due to the complex anatomy of the root canal [7]. Particularly the presence of accessory canals, ramifications and anastomoses may allow residual bacteria to persist, because these areas are often inaccessible to mechanical debridement and chemical irrigation solutions [8]. A RCS with additional antimicrobial properties may therefore not only fill the voids in the radicular dentin through the pressure applied during obturation with gutta-percha, but, at least in theory, may also inhibit growth of any residual bacteria enclosed within the root canal system [9].

Among the clinically available RCS, epoxy resin–based sealers such as AH Plus® (AH) are currently the most used. Various studies have considered AH to be the gold standard RCS, mainly due to its good dimensional stability, low solubility, and resistance to resorption [10]. However, several drawbacks regarding the biocompatibility of AH have been reported, including cytotoxic effects on fibroblasts, potential mutagenic activity, and the induction of severe inflammatory response in bone tissue [11–13]. Concerns have also been raised that AH may exhibit adverse effects on the adjacent host tissues and delay the periapical healing of teeth with AP [14, 15].

In the recent years, several RCS with supposed bioactive properties have been introduced and since then have become the subject of much attention. These novel formulations are claimed to exhibit enhanced biocompatibility and antimicrobial activity, and even support the regeneration of the periapical tissues. BioRoot™ RCS (BR) and TotalFill® BC Sealer (TF) are two calcium silicate–based RCS, whose bioactive features are thought to be attributed to their alkaline pH resulting from calcium hydroxide production and the release of hydroxide ions in an aqueous environment [16]. Calcium hydroxide acts antibacterial by damaging the bacterial membrane and their DNA, but is also known to promote tissue repair by stimulating cell differentiation [17, 18]. These features are beneficial in cases where direct contact to the surrounding tissues is to be expected, such as during treatment of AP, root perforations, root fractures, or root resorptions. GuttaFlow® bioseal (GF) is a bioceramic silicone-based RCS which was introduced in an attempt to combine the filling qualities of gutta-percha and the bioactive properties of calcium silicates in one formulation: fine-grained gutta-percha powder as well as calcium silicate particles are incorporated in the silicone matrix of the sealer. Silicon-based RCS have shown several advantages in previous studies, such as their tight sealing ability, good adaptation to the root canal wall, and low solubility [19–21]. Root canals can be obturated with GF in a cold paste-only filling technique without the additional use of gutta-percha points, and due to its bioactive qualities, it is also considered safe to insert large volumes of GF into the root canal system.

All of these biological benefits seem very promising, but for the bioactive RCS to be a true alternative to epoxy resin–based sealers, they must also satisfy the requirements concerning their physicochemical properties. Specifically, low film thickness and high flowability are important features for a tight seal to prevent bacteria from penetrating the root canal filling.

Since the use of bioactive RCS is becoming more popular among clinicians, there is a need for thorough examinations of the sealers’ biological and physicochemical properties. The main objective of this research is to study the bioactive RCS GF, BR, and TF compared to the gold standard AH focusing on the following parameters: (1) biocompatibility in terms of cytotoxicity and inflammatory response; (2) osteogenic potential; (3) physicochemical properties required for a long-lasting and tight apical seal, such as film thickness, flow, pH, and tightness towards bacterial penetration; (4) potential antimicrobial properties against common endodontic pathogens found in primary root canal infections.

## Materials and methods

### Sealers, cells, and bacterial strains

#### Sealers

In this study, the bioactive endodontic sealers GuttaFlow® bioseal (GF), BioRoot™ RCS (BR) and TotalFill® BC Sealer (TF) were examined and compared to AH plus® (AH). The manufacturers and lot numbers of all sealers are displayed in Table 1.

**Table 1:** Endodontic sealers used in this study and their chemical composition

Root canal sealer (abbr.)	Type	Composition	References	Manufacturer	Lot number
AH plus® (**AH**)	Epoxy-amine resin sealer	Paste A: bisphenol A/F epoxy resin, calcium tungstate, zirconium oxide, silica, iron oxide Paste B: adamantane amine, N,N-dibenzyl-5-oxanonane, TCD-diamine, calcium tungstate, zirconium oxide, silica, silicone oil	[11, 45]	Dentsply Sirona, York, PA, USA	1711001109
GuttaFlow® bioseal (**GF**)	Silicone-based gutta-percha with calcium silicate particles	Gutta-percha, polydimethylsiloxane, zinc oxide, barium sulfate, zirconium oxide, platinum catalyst, color pigments, micro silver, bioactive glass ceramic	[19, 51]	Coltene Holding AG, Altstätten, Switzerland	I67555
BioRoot™ RCS (**BR**)	Calcium silicate sealer	Liquid: aqueous solution of calcium chloride and polycarboxylate powder: tricalcium silicate, zirconium oxide and povidone	[49, 52]	Septodont, Saint-Maur-des-Fossés, France	B20650
TotalFill® BC Sealer (**TF**)	Bioceramic calcium silicate sealer	Zirconium oxide, dicalcium silicate, tricalcium silicate, calcium phosphate monobasic, calcium hydroxide, fillers	[46, 53]	FGK Dentaire, La Chaux-de-Fonds, Switzerland	17004SP

#### Cells

For examining the biological effects of the endodontic sealers, human periodontal ligament fibroblasts (hPDLF, Lonza, Basel, Switzerland), human osteoblasts (hOB, Promocell, Heidelberg, Germany), and human mesenchymal stem cells of male Caucasian origin (hMSC, Lonza) were used. hPDLF and hMSC were cultured in high glucose Dulbecco’s modified eagle’s medium (DMEM, gibco/life technologies), and osteoblast growth medium (OGM, PromoCell GmbH, Heidelberg, Germany) was used for the cultivation of hOB. All media were supplemented with 10% fetal bovine serum (FBS, Merck, Darmstadt, Germany), 100 U/ml of penicillin G and 100 µg/ml streptomycin (Merck). All experiments were performed with cells at passage 4–8 and all cell types were incubated at 37 °C in a humidified atmosphere containing 5% CO_2_.

#### Bacterial strains

Five bacterial species were used for testing the sealers’ antimicrobial properties: the obligate anaerobes *Fusobacterium nucleatum* (ATCC 49256), *Prevotella intermedia* (ATCC 25611), *Parvimonas micra* (ATCC 33270), and *Veillonella parvula* (ATCC 17745) as well as the facultative anaerobe *Streptococcus oralis* (ATCC 35037). All strains were obtained from the German Collection of Microorganisms and Cell Cultures (DSMZ, Braunschweig, Germany) and grown on Schaedler agar plates supplemented with vitamin K_1_ and 5% sheep blood (Becton Dickinson, Franklin Lakes, NJ, USA). Cultivation in liquid medium was performed in anaerobic brain–heart-infusion broth (BHI, Becton Dickinson) supplemented with hemin (5 µg/ml) and vitamin K_1_ (1 µg/ml). The bacteria were incubated at 37 °C in an anaerobic atmosphere (5% H_2_, 10% CO_2_, 85% N_2_).

#### Eluate preparation

Biological effects of the RCS on hPDLF, hOB, and hMSC were examined using their eluates. Sealers were applied to the wells of a 24-well plate (200 mg/well) and exposed to UV light for 30 min for sterilization. The sealers were incubated for 24 h (37 °C, 5% CO_2_, 95% humidity), allowing the material to set. Cell culture medium was added to the wells and the well plates were incubated for either 24 h or 7 days (37 °C, 5% CO_2_, 95% humidity). After incubation, the eluates were sterilized by passing them through a 0.2-µm membrane filter (VWR International GmbH, Darmstadt, Germany) and stored in reaction tubes at − 20 °C until further use.

### Biocompatibility

#### Cytotoxicity

Cytotoxic effects were determined by means of the Colorimetric Cell Viability Kit I (CCVK-I, PromoCell) using the tetrazolium salt WST-8 as an indicator of living cells. Approximately 5 × 10^4^ cells were seeded into the wells of a 96-well plate an incubated overnight. hPDLF and hOB cells were treated with the sealer eluates in different concentrations (undiluted, 1:1 dilution, 1:5 dilution in cell culture medium) as well as with cell culture medium for the control group and incubated for 24 h. CCVK-I was added to the wells according to the manufacturer’s protocol and the absorbance was measured after 120 min of incubation at 450 nm in a spectrophotometer (Varioskan Microplate Reader, Thermo Fisher Scientific, Waltham, MA, USA). Survival rates were computed as a percentage of the control group and the cytotoxic response was rated as non-cytotoxic (> 90% survival), slight (60–90% survival), moderate (30–60% survival), or severe (< 30% survival) [22].

#### Inflammatory response

Interleukin 6 (IL-6) and prostaglandine E_2_ (PGE_2_) release from hPDLF and hOB was used as a parameter for determining the cellular inflammatory response to treatment with the sealer eluates. Adherent hPDLF and hOB in 12-well plates were treated with undiluted sealer eluates for 24 h. Pure cell culture medium served as control group. After incubation, the IL-6 and PGE_2_ levels in the supernatants were measured using a specific enzyme-linked immunosorbent assay (ELISA) according to the manufacturer’s instructions (human IL-6 Quantikine-ELISA Kit, R&D Systems, Minneapolis, MN, USA; PGE_2_ high sensitivity ELISA kit, Enzo life Sciences, Lörrach, Germany). Standard curves were generated to calculate the IL-6 and PGE_2_ concentration. To exclude interference of the ELISA detection kit with the RCS eluates, additional standards were performed with each RCS eluate serving as a diluent.

### Osteogenic potential

For testing the sealers’ ability to induce osteogenic differentiation in progenitor cells, hMSC at passage 4 were seeded in the wells of a 6-well plate and incubated overnight. The cells were treated with 7-day RCS eluates in triplicate as well as with culture medium for the negative control group and incubated for 7 days. Medium supplemented with 100 nM dexamethasone, 10 mM β-glycerophosphate, and 50 µM ascorbic acid (all from Merck, Darmstadt, Germany) served as positive control. The eluates and the medium were changed every 48 h. The cells were washed three times with PBS and fixed with 4% paraformaldehyde solution (Sigma) for 30 min. To determine the amount of calcium deposits within the cells, 40 mM alizarin red S staining solution (ARS, Sigma) was added to each well and incubated for 30 min. After visual examination of the cell cultures, microscopic images of the cells were taken with a phase contrast microscope (Axiovert 40 C, Axiocam 305 color, Carl Zeiss, Oberkochen, Germany). The ARS was then extracted from the cells by adding 10% acetic acid and heating the cell samples for 10 min at a temperature of 85 °C, followed by an incubation on ice for 5 min. After centrifugation for 15 min at 20,000 g, the supernatant was neutralized with 10% ammonium hydroxide and the absorption at 405 nm was determined in a spectrophotometer (Varioskan Microplate Reader). The ARS concentration in the cells was calculated using a standard curve with known concentrations of the dye.

### Physicochemical properties

Film thickness and flow tests were performed in accordance with the DIN EN ISO 6876.

#### Film thickness

Two flat glass plates with a thickness of 5 mm and a contact area of 225 mm^2^ were placed on top of each other and the total thickness of both plates was measured with a digital micrometer (0.1 µm precision). The same measurement was performed with 200 mg of RCS between the glass plates after application of a defined force (150 N) for 10 min. The film thickness of each RCS was determined by calculating the difference in total thickness of both glass plates with and without RCS in between.

#### Flow

Sealers were applied to the center of a glass plate (5 mm thickness). After 180 s, a second glass plate (20 g) was placed on top of the sealer along with a further weight (100 g), measuring a total weight of 120 g. The maximum and minimum diameter of the compressed sealer between the two glass plates was measured. If the difference between the two diameters was not greater than 1 mm, the mean value was calculated.

#### pH

Polytetrafluoroethylene (PTFE) tubes (1.5 mm inner diameter, 10 mm length) were filled with sealers and left to completely set. The PTFE tubes were submerged in falcon tubes containing 10 ml of deionized water. The tubes were incubated at 37 °C and pH measurements were performed after 3 h, 6 h, 9 h, 24 h, 3 days, 7 days, 1 month, and 3 months (827 pH Lab, Deutsche Metrohm GmbH & Co. KG, Filderstradt, Germany).

#### Bacterial penetrability

**Fig. 1:**
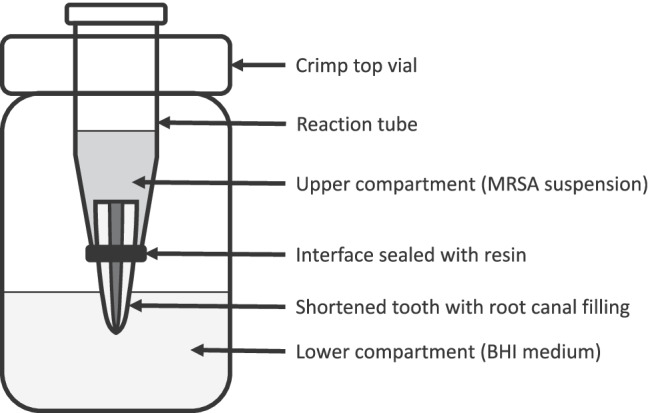
Diagram showing the experimental setup for examining the bacterial penetrability of the RCS

For testing the bacterial penetrability of the endodontic sealers, a previously described method was modified [23]. The use of extracted human teeth was approved by the local ethics committee (registration No. 21–0978 KB). One hundred and four extracted single-rooted, anterior human teeth with mature apex were selected and x-rayed. The coronal portion of all teeth was shortened with a diamond saw to standardize the working length (15 mm). Root canal preparation was performed with a reciprocating single file (Reciproc blue R40, VDW, Munich, Germany). Each root canal was irrigated with 10 ml of 3% sodium hypochlorite (NaOCl), 5 ml of 18% ethylenediaminetetraacetic acid (EDTA), and 2 ml of 0.9% sodium chloride. Subsequently, the teeth were sterilized at 121 °C for 15 min. The teeth were divided into four groups (*n* = 26) for single cone obturation using the four root canal sealers and gutta-percha. Each filled tooth was placed into an individual reaction tube which was cut off at the bottom to expose the tooth apex. The interface between the tooth and the reaction tube was sealed with light-curing flowable resin (SDR flow + , Dentsply Sirona Deutschland GmbH, Bensheim, Germany) and wax. A hole measuring the same diameter of the reaction tube was drilled into the cap of a sterile crimp top vial. The reaction tube with the sealed tooth was placed into the hole, thereby creating two compartments with the filled tooth apex as the only interface. The experimental set-up is shown in Fig. 1. Methicillin-resistant *Staphylococcus aureus* (*MRSA*, DSM 11822) was inoculated in BHI medium supplemented with antibiotics (10 µg/ml amoxicillin; 10 µg/ml ciprofloxacin) and 500 µl of the *MRSA* suspension was added to the reaction tubes (upper compartment). The crimp top vial (lower compartment) was filled with clear BHI medium supplemented with antibiotics, enough to cover 2 mm of the tooth apex in liquid. This set up was incubated for 28 days at 37 °C in a humidified atmosphere containing 5% CO_2_. Once every 4 days, the bacterial suspension in the upper compartment was replaced with a fresh *MRSA* suspension. The medium in the lower compartment was checked for turbidity as a sign of bacterial growth every day. In case the medium of a specimen turned turbid, the day of the event was recorded, and the specimen was not further incubated.

### Antimicrobial properties

For testing the antimicrobial effects of the endodontic sealers, a direct contact test (DCT) was performed by modifying a previously described method [24]. In brief, 200 mg of each sealer were applied to the wells of a 48-well plate and incubated at a temperature of 37 °C and a humidity level of 100% for 24 h, allowing the sealers to set. Overnight cultures of *F. nucleatum*, *P. intermedia*, *P. micra*, *V. parvula*, and *S. oralis* were diluted to an optical density of 0.1 at 600 nm (Varioskan Microplate Reader). The five diluted bacterial suspensions were combined and 750 µl of the multi-species suspension were added to each well containing the dried sealer at the bottom. Blank wells were used for the control group. The well plates were incubated for 48 h in an anaerobic atmosphere. Bacterial growth was observed after 12, 24, and 48 h using a plating and culture method. Colony-forming units (CFU) were determined by serially diluting the bacterial suspensions in 0.9% sodium chloride. The diluted suspensions were plated on agar plates and incubated for 48 h. The CFU were counted following FDA guidelines (only plates with 25 to 250 colonies were considered) and the CFU/ml were calculated. The experiment was performed in duplicate for each experimental condition on five individual days.

### Statistical analyses

All assessments for the biocompatibility and the physicochemical properties were performed in triplicate in independent experiments. Statistical analyses were performed in Python 3.8.0 using *scipy* and *scikit* for inferential statistics and *matplotlib* for the descriptive analysis [25]. The Shapiro–Wilk test revealed normal distribution for all data. Homogeneity of variances was assessed with the Levene’s test. Data with equal variances were analyzed with a one-way analysis of variances and Tukey’s post hoc test. Comparisons between groups for data with unequal variances were performed using Welch’s analysis of variances, followed by a Tamhane’s T2 post hoc test. *P*-values < 0.05 were considered statistically significant. Bacterial penetrability was analyzed by means of a Kaplan–Meier survival analysis using the timeframe from the moment the teeth were incubated in medium until the occurrence of the event of interest (turbidity of the medium). No censored observations occurred during the experiment.

## Results

### Biocompatibility

**Fig. 2:**
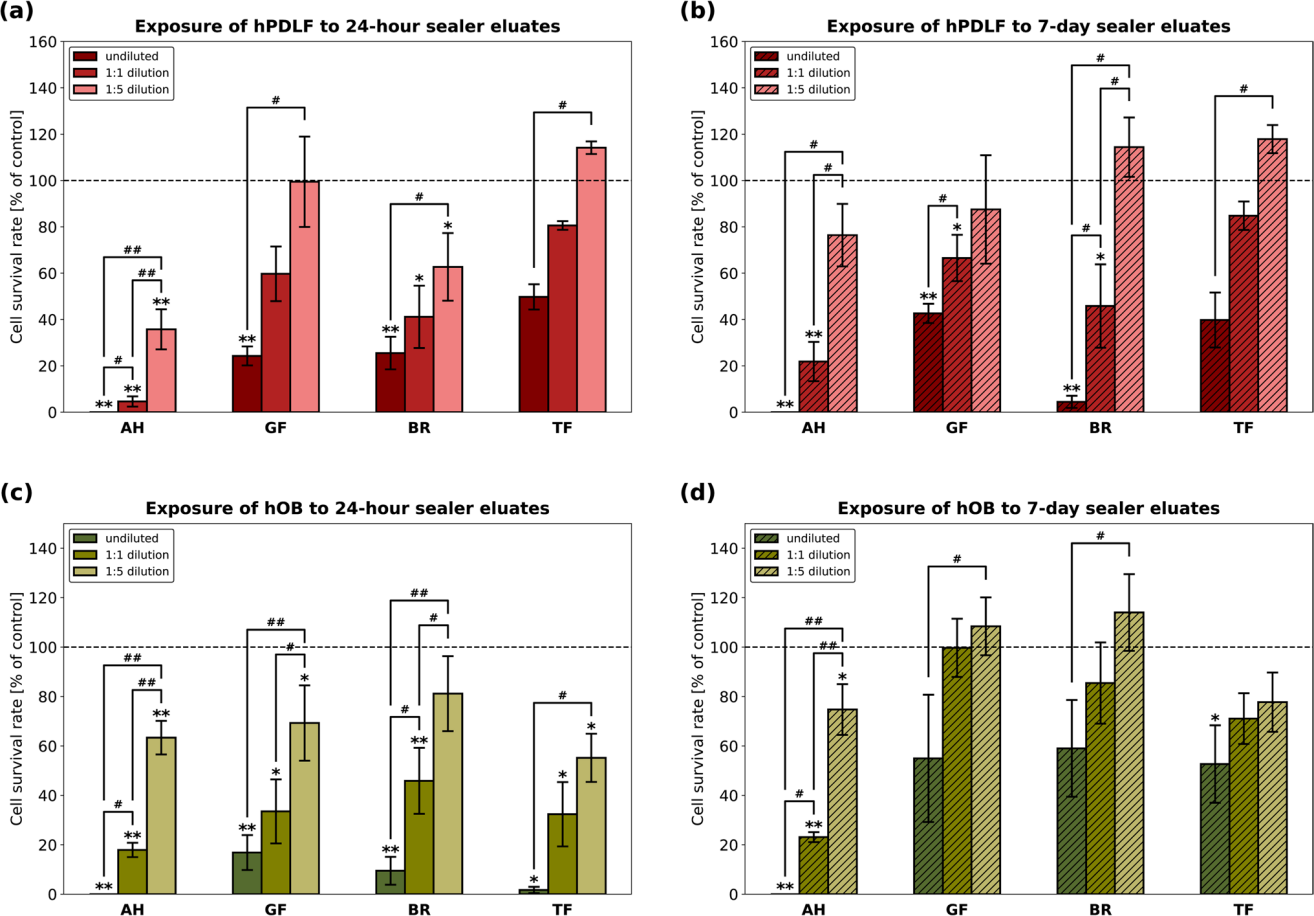
Cell survival rate of hPDLF and hOB after exposure to RCS eluates of AH, GF, BR, and TF. Survival determined with a colorimetric cell viability assay based on the tetrazolium salt WST-8. Data shown as the percentage of the control group. *P*-values determined by Tamhane’s T2 post hoc analysis. *p < 0.05 compared to the control group; **p < 0.001 compared to the control group; ^#^*p* < 0.05 for comparison between two test groups; ^##^*p* < 0.001 for comparison between two test groups

Cytotoxic effects of 24-h and 7-day RCS eluates on hPDLF and hOB were evaluated with a tetrazolium salt reduction assay. Cytotoxicity of the RCS in different dilutions is shown in Fig. 2. Severe toxicity against hPDLF was observed for undiluted 24-h eluates of AH, GF, and BR as well as for undiluted 7-day eluates of AH and BR. 1:5 dilutions of GF and TF eluates were non-toxic to hPDLF. While all undiluted 24-h eluates were severely toxic to hOB, the undiluted 7-day eluates of GF, BR, and TF showed moderate toxicity to hOB. Dilution of the RCS eluates led to an overall reduced cytotoxicity. All diluted sealers were either moderately, slightly, or non-toxic, except for AH eluates, which were still severely toxic to hPDLF and hOB in a 1:1 dilution. A 1:5 dilution significantly reduced the cytotoxic effect of all sealers compared to pure eluates.

**Fig. 3:**
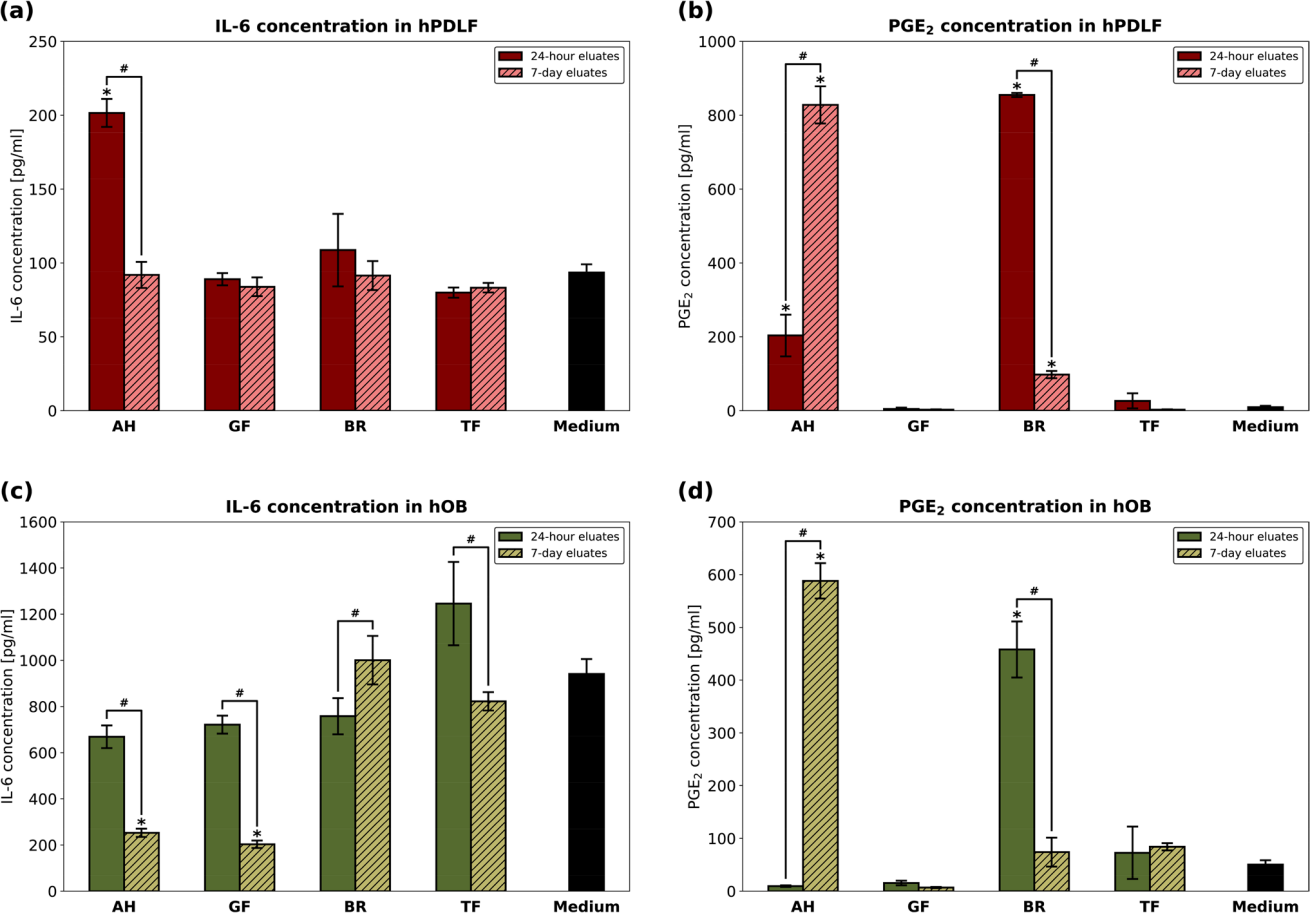
Inflammatory response of hPDLF and hOB to AH, GF, BR, and TF in terms of IL-6 and PGE_2_ levels. Concentrations [pg/ml] measured via specific ELISAs. *P*-values determined by Tamhane’s T2 post hoc analysis. *p < 0.05 compared to the control group; ^#^*p* < 0.05 for comparison between two test groups

Cellular inflammatory response to the RCS eluates in terms of IL-6 and PGE_2_-levels is shown in Fig. 3. The 24-h AH eluates led to the highest IL-6 levels in hPDLF. All other sealer eluates led to similar IL-6 levels as the control group with medium. PGE_2_ concentration in hPDLF was significantly increased in the presence of 7-day AH eluates as well as 24-h BR eluates. The 24-h AH eluates and 7-day BR eluates also increased PGE_2_ levels in hPDLF. IL-6 levels in hOB were similar to the control group for all 24-h eluates and for 7-day eluates of BR and TF. In the presence of AH or GF, the IL-6 concentrations in hOB were significantly reduced compared to the control group. Similar to hPDLF, the PGE_2_ concentrations in hOB were significantly enhanced only when incubated with 7-day AH and 24-h BR eluates.

### Osteogenic potential

**Fig. 4:**
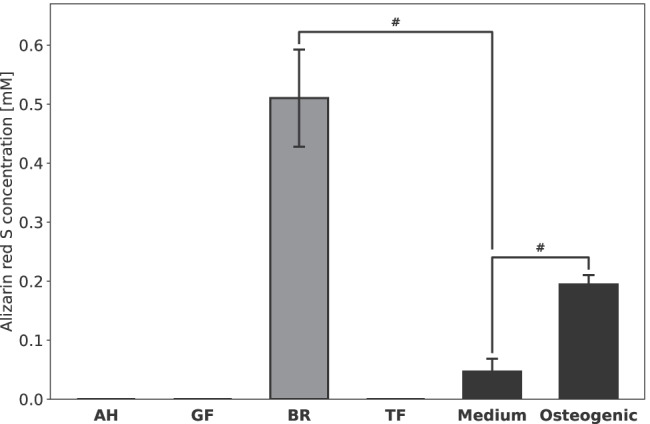
Osteogenic differentiation of hMSC after 7 days exposure to AH, GF, BR, and TF eluates. Data shown in terms of Alizarin red S concentration [mM]. *P*-values determined by Tamhane’s T2 post hoc analysis. ^#^*p* < 0.05 for comparison between two test groups

**Fig. 5:**
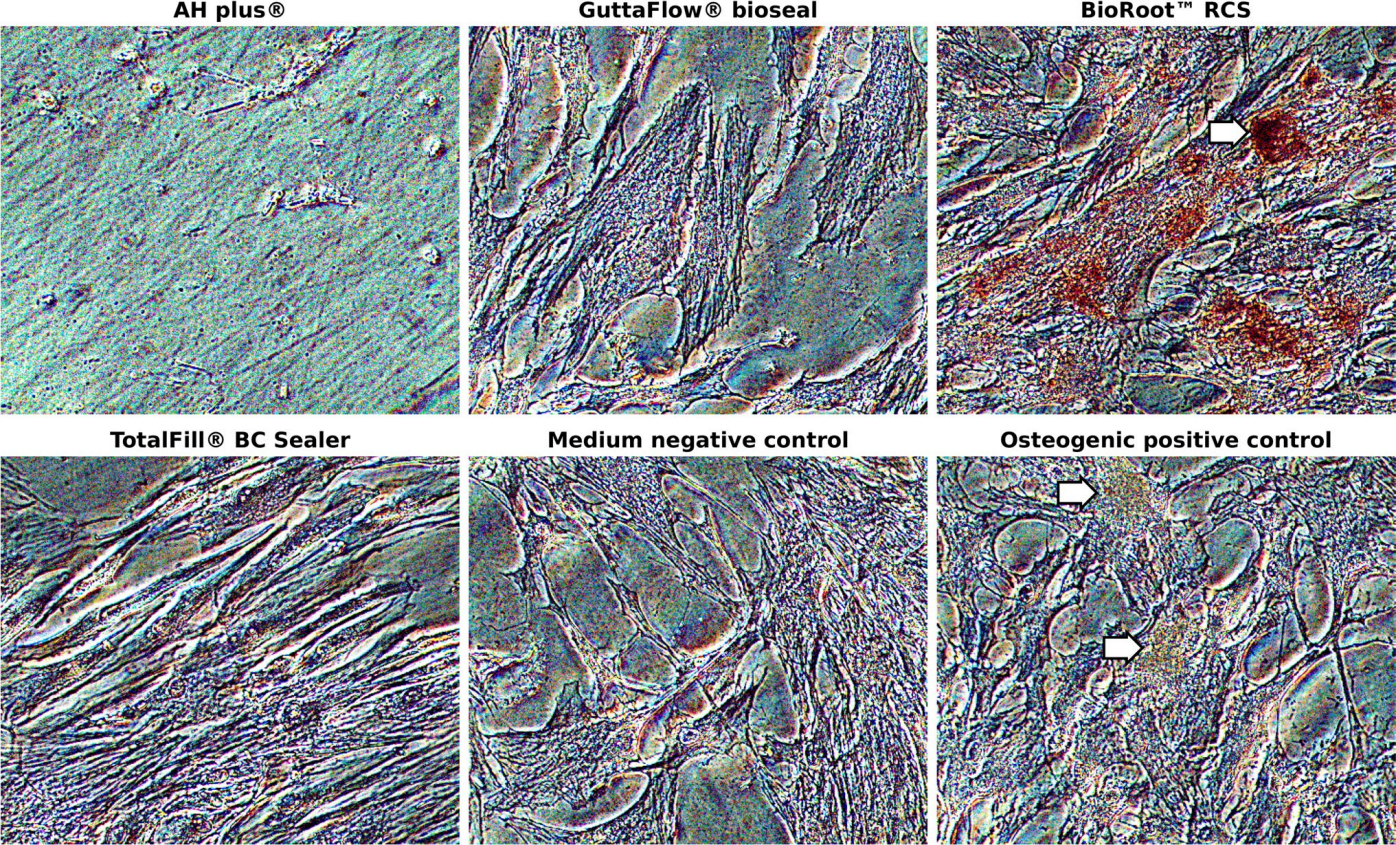
Microscopic images of hMSC after exposure to different RCS for 7 days (10 × magnification). Cells were stained with Alizarin red S staining solution to visualize cellular calcium deposits as an indicator of osteogenic differentiation. Arrows indicate possible nodules of mineralization

The results for the sealer’s ability to induce osteogenic differentiation in hMSC are displayed in Fig. 4 in terms of the ARS concentration. Only BR led to a significant increase in ARS concentration. Microscopic images of the cells after staining with ARS are shown in Fig. 5. The calcium deposits in hMSC (stained red) after incubation with BR are distinctly visible. The presence of AH led to total cell death and caused the cells to detach from the surface. For hMSC incubated with GF or TF, no calcium deposits were observed after ARS staining, but they showed a similar cell morphology to the negative control group with cell culture medium.

### Physicochemical properties

The results for film thickness and flow are shown in Table 2. The film thicknesses of TF, AH, and GF were similar and fulfilled the requirements of the ISO 6876 standard (film thickness < 50 µm). The film thickness value for BR was larger than the maximum film thickness required by the ISO standard. The film thickness values in increasing order were TF < AH < GF < BR.

**Table 2:** Physical properties of the endodontic sealers used in this study. Data shown as means and standard deviation

	**AH**	**GF**	**BR**	**TF**
Film thickness [µm]	26.33 (± 1.53)	27.33 (± 1.53)	67.67 (± 6.66)	25.00 (± 1.00)
Flowability [mm]	24.33 (± 1.23)	16.02 (± 0.28)	21.67 (± 1.26)	26.28 (± 1.33)

TF presented the highest flowability among all tested sealers. GF showed the lowest flowability and was the only RCS which did not meet the requirements of the ISO standard (diameter > 17 mm). The diameters indicating the flowability in decreasing order were TF > AH > GF > BR.

**Fig. 6:**
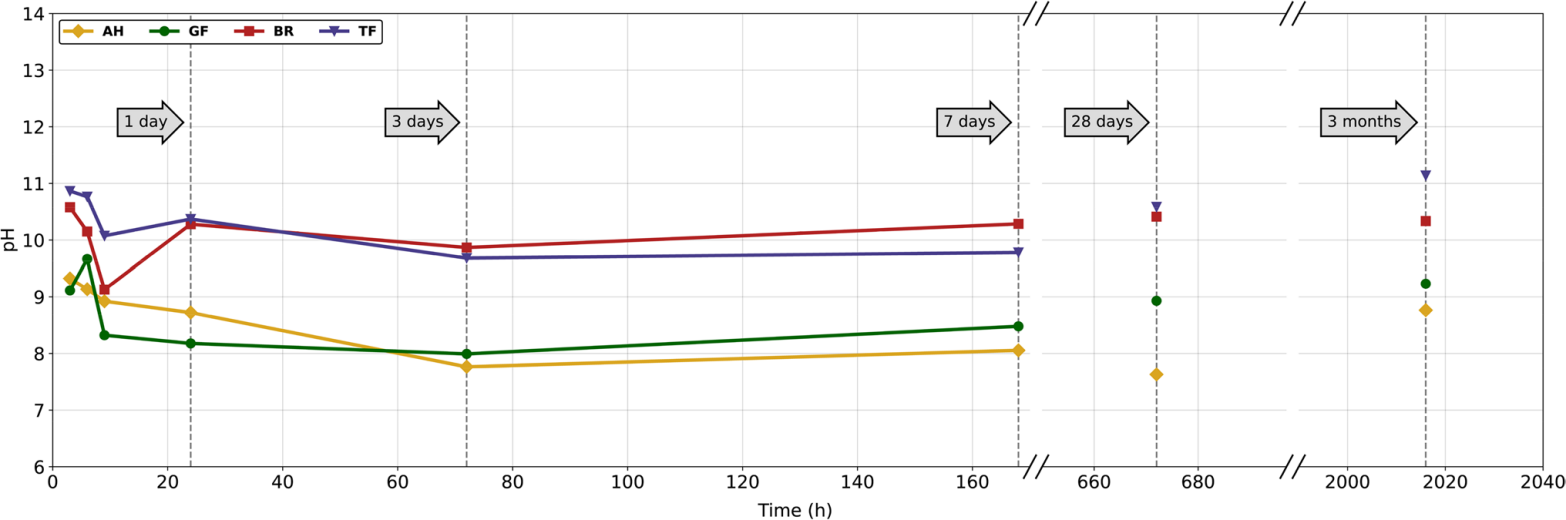
pH changes of deionized water containing AH, GF, BR, and TF specimens over a total observation time of 3 months

Changes in 9 are shown in Fig. 6. For all four sealers, an initial drop in pH within the first 9 h was observed. The pH of BR increased after 24 h and remained stable for the rest of the experiment, whereas AH, GF, and TF started to show a slight increase in pH only after 3 months. TF and BR were the most alkaline for the entire experimental period.

**Fig. 7:**
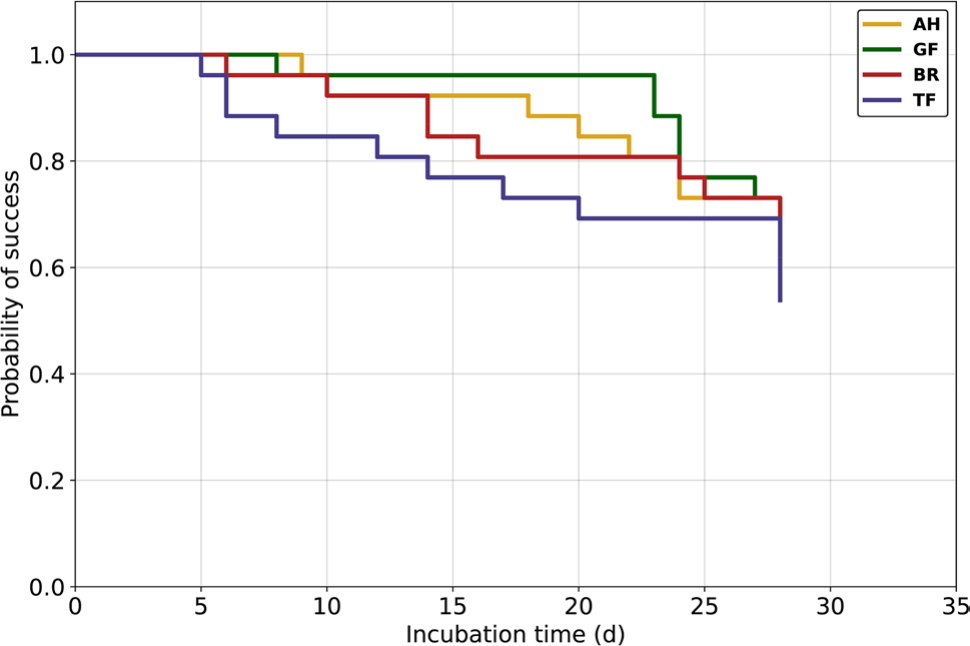
Kaplan–Meier survival analysis of human extracted teeth filled with AH, GF, BR, and TF shows the occurrence of bacterial penetration at each point in days. No censored observations occurred during the experiment

Figure 7 shows the success estimate for the probability of bacterial penetration of the sealers in a Kaplan–Meier survival analysis. The overall success rate after an observation period of 28 days was 62% for AH and BR, 58% for GF and 54% for TF.

### Antimicrobial efficacy against endodontic pathogens 

**Fig. 8:**
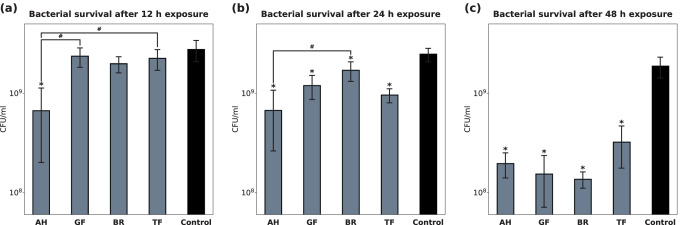
Antimicrobial effects of AH, GF, BR, and TF against a multi-species suspension containing bacterial cultures of *F. nucleatum*, *P. intermedia*, *P. micra*, *V. parvula*, and *S. oralis* in equal amounts (no-agent control group with BHI medium). Bacterial survival is shown in terms of CFU/ml after an incubation time of 12, 24, and 48 h. *P*-values determined by Tukey’s HSD post hoc analysis. *p < 0.05 compared to the control group; ^#^*p* < 0.05 for comparison between two test groups

Antimicrobial effects of the RCS were assessed in a modified DCT using a multi-species bacterial suspension with common endodontic pathogens. Bacterial survival after 12, 24, and 48 h exposure to the RCS is shown in Fig. 8. AH was the first to show significant antibacterial activity after 12 h, but after 24 h of incubation, the presence of all RCS reduced the CFU/ml significantly. The mean bacterial growth compared to the control group was 10.46% for AH, 8.21% for GF, 7.27% for BR, and 17.21% for TF after an exposure time of 48 h.

## Discussion

Bioactive RCS are thought to be extremely biocompatible and actively support the regeneration of tissues surrounding the root canal such as the periapical region and the periodontal ligament. Aside from having these features, it is necessary for an ideal RCS to provide good physical and chemical properties and show antimicrobial activity against endodontic pathogens. This study examined a broad set of clinically relevant features of three bioactive endodontic RCS. An epoxy resin–based RCS was used as a comparison, since it is still considered the gold standard in endodontics.

For evaluating the cytotoxicity of dental materials, in vitro toxicological examinations are frequently performed using various cell types, including murine or hamster cells. Although there are many similarities between human and animal cells, the number of protein-coding genes can be very different depending on the species, which may influence the interpretation of cytotoxic evaluations. Thus, in this study, we chose to perform all biological examinations with representative cell types, such as cells of the periodontal ligament and osteoblasts, both of human origin, assuming that RCS applied to the root canal of teeth with AP are in proximity to the periodontal ligament and bone tissue at the tooth apex. Furthermore, we aimed to examine initial toxic effects of the RCS by using eluates extracted after 24 h of incubation as well as their late toxic effects after allowing the components released from the RCS to accumulate for 7 days within the eluates. With these two elution methods we were also able to judge the chemical stability of any potentially toxic components released by the RCS. Given that in vivo RCS eluates are diluted when they are exposed to tissue fluid and exudate at the tooth apex, the WST-8 reduction assay was performed with RCS eluates both in their pure form as well as in different dilutions. Our results indicate that all four RCS display moderate-to-severe initial toxicity to hPDLF and hOB; however, AH was the only RCS which left no cells to survive after exposure to pure AH eluates. A 1:1 dilution reduced the toxicity of all bioactive RCS to hPDLF and hOB, while AH was still severely toxic to both cell types. AH was also found to be generally cytotoxic regardless of the tested cell line. During the osteogenic differentiation experiment, which was performed using hMSC, we regularly inspected the cells under a light microscope and observed that the presence of AH caused the cells to detach from the culture surface after 2 days of incubation. This finding marks cell death and also confirms our toxicity results obtained from the WST-8 reduction assay. Furthermore, our results are consistent with data from previous studies which have reported severe toxicity of AH against osteoblasts and periodontal ligament fibroblasts, while GF, BR, and TF were shown to be less cytotoxic than AH [26–30]. As far as differences in toxicity of the 24-h and 7-day RCS eluates is concerned, our results revealed an overall lower cytotoxicity of the 7-day eluates, especially when diluted. This finding suggests that the toxic components released during the first 24 h of elution exhibit a relatively low molecular stability, which may have led to a decline in toxic activity of the RCS after only a few days. This would be favorable for all RCS, especially since the diluted eluates are more representative of their long-term cytotoxicity, whereas the undiluted eluates can be viewed as the worst-case scenario because they contain all eluted compounds in higher concentrations.

Inflammation as a host reaction to toxic stimuli is characterized by the release of numerous chemical mediators, including cytokines, histamines, and prostaglandins. IL-6 is an important proinflammatory cytokine which is promptly produced by various cell types in response to local tissue injuries and infections, marking the initial stage of inflammation [31]. IL-6 is known to cause an increase in matrix-metalloproteinase-1 (MMP-1), which destroys connective tissue by either directly degrading collagen or by activating the fibrinolytic protease cascade [32]. Elevated levels of IL-6 have also been found to be associated with symptomatic and larger periapical lesions [33, 34]. PGE_2_ is a further proinflammatory mediator which is synthesized from arachidonic acid by the cyclooxygenases COX-1 and COX-2 and the PGE synthases. The abundance of PGE_2_ in periapical tissues is associated with elevated pain sensation caused by PGE_2_-induced vasodilatation and increased vascular permeability [33]. Both IL-6 and PGE_2_ have been identified as mediators of bone resorption and have been found to promote resorption of the alveolar bone in patients with periodontitis by stimulating the osteoclastic activity [35]. However, increased cytokine secretion is not an unfavorable circumstance by definition. While PGE_2_ at high concentrations has been proven to support bone resorption, there is evidence that at low concentrations PGE_2_ also promotes bone formation by stimulating DNA and collagen synthesis in osteoblasts [36]. Our in vitro data show that IL-6 levels in hPDLF were significantly increased in the presence of the 24-h AH eluates, while all other eluates led to IL-6 concentrations similar to those of the control group. However, in hOB, the production of IL-6 was reduced by 7-day AH and GF eluates. AH and BR severely stimulated the PGE_2_ expression in hPDLF and hOB, but BR led to enhanced PGE_2_ production only when eluted for 24 h and displayed a weaker stimulatory effect with 7-day eluates. This finding was vice versa for AH, as we observed a more severe secretion of PGE_2_ when AH was eluted for 7 days. When interpreting the cytokine release from cells, the total number of viable cells capable of producing IL-6 and PGE_2_ during exposure to the RCS eluates have to be considered. Given that the RCS exerted cytotoxic effects as previously analyzed with the WST-8 reduction assay, there were probably also fewer cells secreting cytokines. However, both IL-6 and PGE_2_ are stable in vitro, and therefore, the ELISA probably also detected any cytokines which were secreted by the cells prior to their death [37, 38]. Another condition affecting the cellular inflammatory reaction is the mode of cell death because the presence of cytoplasmic contents in the extracellular space incites an inflammatory reaction in the remaining cells. However, this circumstance is only to be found in 2 undergoing necrosis, where the membrane integrity is disturbed and cytoplasmic shedding occurs, whereas in apoptotic cell bodies, the cell membrane remains intact, and the cytoplasm is retained within the cells [39]. Nonetheless, we cannot draw certain conclusions about the biological interactions solely based on our cell viability and inflammation data, because we are lacking parameters, such as when the cells died or whether they died due to apoptosis or necrosis. Furthermore, it is important to bear in mind that in vitro experiments do not replicate the conditions of cells in an organism, where such biological interactions are far more complex and several immune cells are present to manage the inflammation. This limits the significance of our data to predict possible in vivo inflammation caused by the RCS.

One of the alleged properties of bioactive RCS is their ability to support the osseous healing of the periapical region. Therefore, we tested their osteogenic potential by incubating hMSC with RCS eluates for 7 days and quantifying cellular calcium deposits by means of an ARS staining protocol. Calcium content within the cells indicates mineralization and hence the osteogenic differentiation of the progenitor cells [40]. BR was the only RCS which led to nodule-shaped areas that were stained by the ARS staining solution, suggesting signs of possible cellular calcium uptake. However, we cannot be certain if these calcium deposits were solely caused by osteogenic differentiation. Since BR as well as the other bioactive RCS contain di- or tricalcium silicates, it is possible that the stained areas were also due to calcium compounds released from the RCS which enriched in the cell layers as well. Measuring the calcium content as an indicator of osteogenic differentiation may therefore not be the assay of choice when dealing with eluates from materials containing calcium. This limits the significance of these results, especially in view of the poorly discernable mineralization in the positive control, where the calcium nodules were not stained in a deep red color as expected but merely showed a yellow tint. Therefore, further investigations are necessary to verify the true osteogenic potential of bioactive RCS.

In addition to the cytotoxic and osteogenic effects of the RCS, this study also addressed their antimicrobial activity in connection with their physicochemical properties. Since primary root canal infections are typically polymicrobial, anaerobic infections, a combination of different bacteria was used rather than just one species for testing the antimicrobial effects of the RCS. Previous studies have shown that oral streptococci, *Fusobacterium* spp., *Prevotella* spp., and *P. micra* are among the most prevalent species in primary root canal infections [41–43]. Our results revealed that after 12 h of incubation, AH was the first and only RCS which significantly reduced the number of bacteria in the multi-species suspension. For all other RCS, the first significant bactericidal activity was recorded after 24 h. After 48 h of incubation, the bacterial reduction was similar among all of the tested RCS. No significant difference between the four groups was observed after 48 h. However, the overall antimicrobial efficacy of all four RCS is to be rated as moderate or low, considering that the mean CFU/ml dropped by just 0.5–1 log-scales. These findings are also consistent with data from previous studies, although none of these studies assessed the antibacterial effects using multi-species bacterial suspensions [30, 44, 45]. Furthermore, there is evidence from our in vitro results that the antimicrobial efficacy of the RCS may be different depending on the bacterial species. Using a culture method for quantifying the number of bacteria allowed us to also distinguish the five bacterial species on the agar plates based on their different morphology. Thus, we were able to roughly judge to which extent the growth of each bacterial species was inhibited by the RCS. An example agar plate of the control group showing the cultured multi-species suspension is displayed as supplementary information (SI1). In general, *S. oralis* colonies were highest in number on all agar plates of the RCS groups, whereas *P. intermedia* seemed to have barely survived in the presence of AH, GF, and BR. Notably more *P. intermedia* colonies were visible on the agar plates of the TF group, suggesting a lower efficacy of TF against this bacterial species. However, to be certain about the specific antibacterial efficacy against each bacterial species, the DCT would have to be performed using separate mono-species suspensions instead of a multi-species mix.

Sufficient tightness of the root canal filling and the coronal restoration is a prerequisite for long-term treatment success in endodontics [1]. If at least one of both conditions is not provided, there is a higher risk for microleakage through the filling material, allowing bacteria to recolonize the root canal system and cause persistent infections. We examined the tightness of the RCS towards bacterial penetration using MRSA and a culture medium containing antibiotics to avoid cross-contamination and allow bacterial growth only for the species which was used for inoculating the medium in the upper compartment. The extracted teeth were prepared, disinfected, and then obturated in single cone technique using gutta-percha and each of the four tested RCS. For blocking the dentinal tubes and accessory canals during obturation, low film thickness and high flowability of the RCS are preferable features. Our evaluations showed that these two features were best in AH and TF; however, the first event of failure in the bacterial penetrability experiment occurred in the TF group after 5 days of incubation. The lowest overall survival rate after 30 days was registered for the TF group as well. Initial failure occurred last in the AH group, and the best overall survival rate after 30 days was observed for AH. Despite having a similar film thickness and flowability, the root canal fillings with AH and TF seemed to have varied in tightness. This finding can be explained by further physical properties that were not part of this research. Data from a previous study suggest that TF has a higher solubility and a lower dimensional stability than AH, which may have impaired the tightness of the root canal filling during the 30 days of the experiment [46]. Specifically, TF showed a 7% mass loss after 7 days and a 13% mass loss after 30 days. The solubility of AH on the other hand was very low since only a 0.4% mass loss was recorded after 30 days. These findings are also supported by further studies, which have proved TF to have a low dimensional stability [30, 47]. While BR has also been shown to be more soluble than AH, GF is the only RCS which has a reportedly lower solubility than AH, which could probably be due to its polydimethylsiloxane component [48–50]. These results are also consistent with our observations made for the bacterial penetrability of the RCS tested in this study. Besides initial failure of one specimen on day 8, no further specimen in the GF group turned turbid until day 23. The GF group therefore showed the longest period in which no event of failure occurred. Naturally, the observation time of 30 days presents a limitation of this experiment. Considering that a root canal filling ideally remains within the tooth for many years, it would be interesting to observe the in vitro tightness of root canal fillings in an extended time frame, perhaps until more than 90% of the specimens have failed.

## Conclusions

Within the limitations of an in vitro study, we were able to show favorable results regarding the biocompatibility of the tested bioactive RCS GF, BR, and TF. All RCS exhibited similar antimicrobial properties against common endodontic pathogens in primary root canal infections. The overall antimicrobial efficacy was moderate since none of the tested RCS achieved a bacterial reduction greater than one log scale. Nonetheless, the bioactive RCS may be a useful alternative to epoxy resin–based RCS, for example, in cases where the RCS may come into direct contact with viable cells of the surrounding tissues, such as during treatment of teeth with AP, for repairing root perforations, or during endodontic surgery. However, the results of an in vitro study must be considered carefully and may not be directly applied to clinical practice, because biological interactions and healing procedures are far more complex in a living organism and cannot be replicated in laboratory experiments. Therefore, there is a need for in vivo studies which should focus, for example, on providing long-term clinical data on the recovery of teeth that have been treated with different bioactive RCS.

## Supplementary Information

Below is the link to the electronic supplementary material.

Supplementary file1 (PDF 1578 KB)

## References

[1] Ray HA, Trope M (1995). Periapical status of endodontically treated teeth in relation to the technical quality of the root filling and the coronal restoration. Int Endod J.

[2] Löst C (2006). Quality guidelines for endodontic treatment: consensus report of the European Society of Endodontology. Int Endod J.

[3] Ricucci D, Russo J, Rutberg M (2011). A prospective cohort study of endodontic treatments of 1,369 root canals: results after 5 years. Oral Surg, Oral Med Oral Pathol Oral Radiol Endodontol.

[4] Siqueira JF, Rôças IN, Riche FNSJ, Provenzano JC (2008). Clinical outcome of the endodontic treatment of teeth with apical periodontitis using an antimicrobial protocol. Oral Surg Oral Med Oral Pathol Oral Radiol Endodontol.

[5] Ng YL, Mann V, Gulabivala K (2011). A prospective study of the factors affecting outcomes of nonsurgical root canal treatment: Part 1: Periapical health. Int Endod J.

[6] Bouillaguet S, Manoil D, Girard M (2018). Root microbiota in primary and secondary apical periodontitis. Front Microbiol.

[7] Trope M, Debelian G (2009). Microbial control: the first stage of root canal treatment. Gen Dent.

[8] Nair PNR (2006). On the causes of persistent apical periodontitis: a review. Int Endod J.

[9] Mohammadi Z, Dummer PMH (2011). Properties and applications of calcium hydroxide in endodontics and dental traumatology. Int Endod J.

[10] McMichen FRS, Pearson G, Rahbaran S, Gulabivala K (2003). A comparative study of selected physical properties of five root-canal sealers. Int Endod J.

[11] Schweikl H, Schmalz G, Federlin M (1998). Mutagenicity of the root canal sealer AHPlus in the Ames test. Clin Oral Investig.

[12] Cohen BI, Pagnillo MK, Musikant BL, Deutsch AS (2000). An in vitro study of the cytotoxicity of two root canal sealers. J Endod.

[13] Sousa CJA, Montes CRM, Pascon EA (2006). Comparison of the Intraosseous biocompatibility of AH Plus, EndoREZ, and epiphany root canal sealers. J Endod.

[14] Khandelwal A, Jose J, Teja K-V, Palanivelu A (2022). Comparative evaluation of postoperative pain and periapical healing after root canal treatment using three different base endodontic sealers - a randomized control clinical trial. J Clin Exp Dent.

[15] Sari S, Duruturk L (2007). Radiographic evaluation of periapical healing of permanent teeth with periapical lesions after extrusion of AH Plus sealer. Oral Surg Oral Med Oral Pathol Oral Radiol Endod.

[16] Ørstavik D (2005). Materials used for root canal obturation: technical, biological and clinical testing. Endod Top.

[17] Lohbauer U, Gambarini G, Ebert J (2005). Calcium release and pH-characteristics of calcium hydroxide plus points. Int Endod J.

[18] Siqueira JFJ, Lopes HP (1999). Mechanisms of antimicrobial activity of calcium hydroxide: a critical review. Int Endod J.

[19] Gandolfi MG, Siboni F, Prati C (2016). Properties of a novel polysiloxane-guttapercha calcium silicate-bioglass-containing root canal sealer. Dent Mater.

[20] Elayouti A, Achleithner C, Löst C, Weiger R (2005). Homogeneity and adaptation of a new gutta-percha paste to root canal walls. J Endod.

[21] Brackett MG, Martin R, Sword J (2006). Comparison of seal after obturation techniques using a polydimethylsiloxane-based root canal sealer. J Endod.

[22] de Rossato TC, A, Gallas JA, da Rosa WLO,  (2017). Experimental sealers containing metal methacrylates: physical and biological properties. J Endod.

[23] Torabinejad M, Rastegar AF, Kettering JD, Pitt Ford TR (1995). Bacterial leakage of mineral trioxide aggregate as a root-end filling material. J Endod.

[24] Heyder M, Kranz S, Völpel A (2013). Antibacterial effect of different root canal sealers on three bacterial species. Dent Mater.

[25] van Rossum G, Drake F (2019) Python 3 Reference Manual

[26] Jung S, Sielker S, Hanisch MR (2018). Cytotoxic effects of four different root canal sealers on human osteoblasts. PLoS ONE.

[27] Szczurko G, Pawińska M, Łuczaj-Cepowicz E (2018). Effect of root canal sealers on human periodontal ligament fibroblast viability: ex vivo study. Odontology.

[28] Huang FM, Tai KW, Chou MY, Chang YC (2002). Cytotoxicity of resin-, zinc oxide-eugenol-, and calcium hydroxide-based root canal sealers on human periodontal ligament cells and permanent V79 cells. Int Endod J.

[29] Collado-Gonzalez M, Tomas-Catala CJ, Onate-Sanchez RE (2017). Cytotoxicity of GuttaFlow Bioseal, GuttaFlow2, MTA Fillapex, and AH Plus on human periodontal ligament stem cells. J Endod.

[30] Colombo M, Poggio C, Dagna A (2018). Biological and physico-chemical properties of new root canal sealers. J Clin Exp Dent.

[31] Tanaka T, Narazaki M, Kishimoto T (2014). IL-6 in inflammation, immunity, and disease. Cold Spring Harb Perspect Biol.

[32] Naruishi K, Nagata T (2018). Biological effects of interleukin-6 on gingival fibroblasts: cytokine regulation in periodontitis. J Cell Physiol.

[33] Martinho FC, Chiesa WMM, Leite FRM (2012). Correlation between clinical/radiographic features and inflammatory cytokine networks produced by macrophages stimulated with endodontic content. J Endod.

[34] Gazivoda D, Dzopalic T, Bozic B (2009). Production of proinflammatory and immunoregulatory cytokines by inflammatory cells from periapical lesions in culture. J Oral Pathol Med.

[35] McCauley LK, Nohutcu RM (2002). Mediators of periodontal osseous destruction and remodeling: principles and implications for diagnosis and therapy. J Periodontol.

[36] Watrous DA, Andrews BS (1989). The metabolism and immunology of bone. Semin Arthritis Rheum.

[37] Kalinski P (2012). Regulation of immune responses by prostaglandin E2. J Immunol.

[38] De Jager W, Bourcier K, Rijkers GT (2009). Prerequisites for cytokine measurements in clinical trials with multiplex immunoassays. BMC Immunol.

[39] Elmore S (2007). Apoptosis: a review of programmed cell death. Toxicol Pathol.

[40] Lee B-N, Hong J-U, Kim S-M (2019). Anti-inflammatory and osteogenic effects of calcium silicate-based root canal sealers. J Endod.

[41] Rôças IN, Siqueira JF (2012). Antibiotic resistance genes in anaerobic bacteria isolated from primary dental root canal infections. Anaerobe.

[42] Kist S, Kollmuss M, Jung J (2017). Comparison of ozone gas and sodium hypochlorite/chlorhexidine two-visit disinfection protocols in treating apical periodontitis: a randomized controlled clinical trial. Clin Oral Investig.

[43] Siqueira JFJ, Rôças IN (2008). Clinical implications and microbiology of bacterial persistence after treatment procedures. J Endod.

[44] Alsubait S, Albader S, Alajlan N (2019). Comparison of the antibacterial activity of calcium silicate- and epoxy resin-based endodontic sealers against *Enterococcus faecalis* biofilms: a confocal laser-scanning microscopy analysis. Odontology.

[45] Ruiz-Linares M, Baca P, Arias-Moliz MT (2019). Antibacterial and antibiofilm activity over time of guttaflow bioseal and AH plus. Dent Mater J.

[46] Tanomaru-Filho M, Torres FFE, Chávez-Andrade GM (2017). Physicochemical properties and volumetric change of silicone/bioactive glass and calcium silicate–based endodontic sealers. J Endod.

[47] Poggio C, Dagna A, Ceci M (2017). Solubility and pH of bioceramic root canal sealers: a comparative study. J Clin Exp Dent.

[48] Urban K, Neuhaus J, Donnermeyer D (2018). Solubility and pH value of 3 different root canal sealers: a long-term investigation. J Endod.

[49] Siboni F, Taddei P, Zamparini F (2017). Properties of bioroot RCS, a tricalcium silicate endodontic sealer modified with povidone and polycarboxylate. Int Endod J.

[50] Zhou HM, Shen Y, Zheng W (2013). Physical properties of 5 root canal sealers. J Endod.

[51] Saygili G, Saygili S, Tuglu I, Capar ID (2017). In vitro cytotoxicity of guttaflow bioseal, guttaflow 2 AH-Plus and MTA fillapex. Iran Endod J.

[52] Reszka P, Nowicka A, Lipski M, et al (2016) A comparative chemical study of calcium silicate-containing and epoxy resin-based root canal sealers. Biomed Res Int 2016. 10.1155/2016/9808432 10.1155/2016/9808432 PMC5206425 28097154

[53] Lim M, Jung C, Shin D-H (2020). Calcium silicate-based root canal sealers: a literature review. Restor Dent Endod.

