# Supplementary material for: A comprehensive in vitro comparison of the biological and physicochemical properties of bioactive root canal sealers
Source: Clin Oral Investig. 2022 Jun 3;26(10):6209–22. doi: 10.1007/s00784-022-04570-2 (PMC9525420; doi:10.1007/s00784-022-04570-2)
Supplement: Supplementary file 1 — Supplementary file1 (PDF 1578 KB) [file 784_2022_4570_MOESM1_ESM.pdf]

SUPPLEMENTARY INFORMATION

Do the biological and physicochemical properties of bioactive root canal sealers support the periapical regeneration? – a comparative *in vitro* study

Journal: **Clinical Oral Investigations**

*Sabina Noreen **WUERSCHING**[a]
Christian **DIEGRITZ**[a]
Reinhard **HICKEL**[a]
Karin Christine **HUTH**[a]
Maximilian **KOLLMUSS**[a]

[a] Department of Conservative Dentistry and Periodontology, University Hospital, LMU Munich, Goethestrasse 70, 80336 Munich, Germany

* Corresponding Author
Sabina Noreen **WUERSCHING**, DDS
Department of Conservative Dentistry and Periodontology
University Hospital, LMU Munich
Goethestrasse 70
80336 Munich, Germany
+49 89 4400 59307
sabina.wuersching@med.uni-muenchen.de

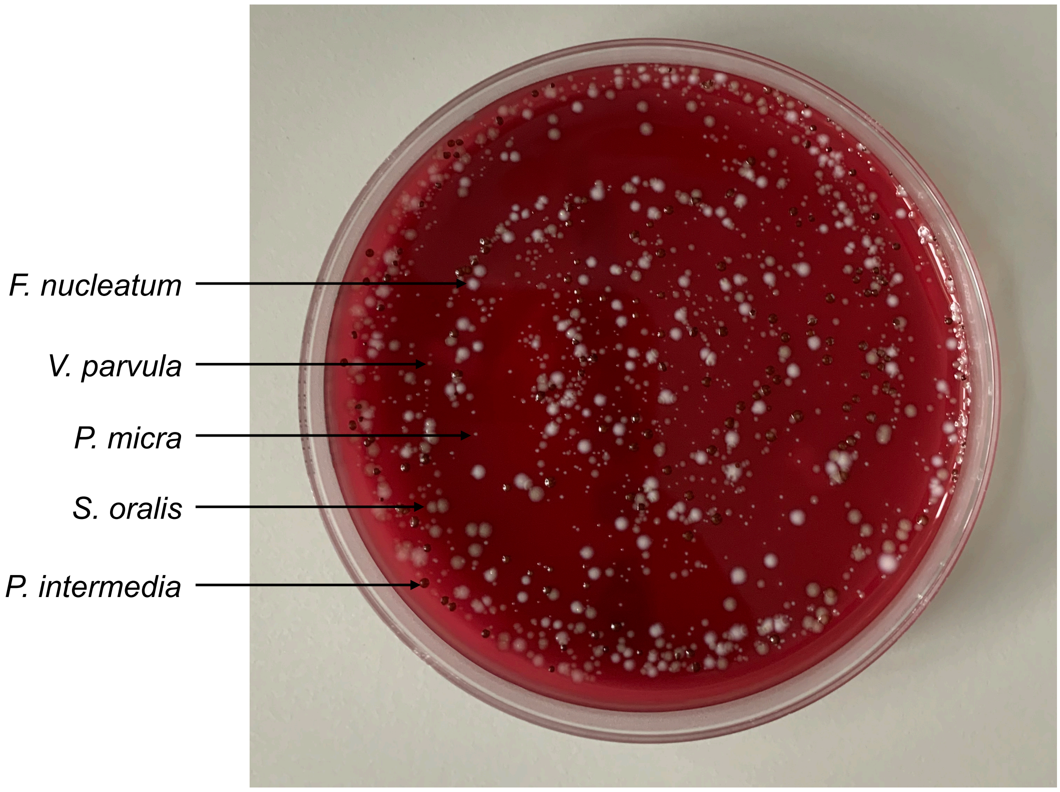

SI 1 Example agar plate of a control group showing the five different bacterial species
